# Primary tumor resection improves prognosis of unresectable carcinomas of the transverse colon including flexures with liver metastasis: a preliminary population-based analysis

**DOI:** 10.1186/s12885-021-08157-0

**Published:** 2021-05-06

**Authors:** Jiefeng Zhao, Jinfeng Zhu, Rui Sun, Chao Huang, Rongfa Yuan, Zhengming Zhu

**Affiliations:** 1 Department of Gastrointestinal Surgery, The Second Affiliated Hospital of Nanchang University, No. 1, Minde Road, Nanchang, Jiangxi 330006 People’s Republic of China; 2 Department of Hepatobiliary and Pancreatic Surgery, The Second Affiliated Hospital of Nanchang University, No. 1, Minde Road, Nanchang, Jiangxi 330006 People’s Republic of China

**Keywords:** Colorectal cancer liver metastasis, Transverse colon, Hepatic flexure, Splenic flexure, Primary tumor resection, Survival, SEER

## Abstract

**Purpose:**

Studies on unresectable colorectal cancer liver metastasis(CRLM) rarely analyze the prognosis of the patients from the point of colonic subsites. We aimed to evaluate the effect of primary tumor resection (PTR) and different scope of colectomy on the prognosis of patients with unresectable transverse colon cancer liver metastasis (UTCLM), hepatic flexure cancer liver metastasis (UHFLM), and splenic flexure cancer liver metastasis (USFLM).

**Patients and methods:**

The patients were identified from the Surveillance, Epidemiology, and End Results (SEER) database from 2010 to 2015. Cox proportional hazards regression models were used to identify prognostic factors of overall survival (OS) and cause-specific survival (CSS). Kaplan-Meier analyses and log-rank tests were conducted to assess the effectiveness of PTR on survival.

**Results:**

In total, this study included a cohort of 1960 patients: 556 cases of UHFLM, 1008 cases of UTCLM, and 396 cases of USFLM. The median survival time of whole patients was 11.0 months, ranging from 7.0 months for UHFLM patients to 15.0 months for USFLM patients. USFLM patients had the best OS and CSS, followed by UTCLM patients. UHFLM patients had the worst OS and CSS (All *P* < 0.001). PTR could improve the OS and CSS of UTCLM, UHFLM, and USFLM (All *P* < 0.001). Subgroups analysis revealed that USFLM patients with tumor size≤5 cm and negative CEA had not demonstrated an improved OS and CSS after PTR. Multivariate analysis showed that PTR and perioperative chemotherapy were common independent prognostic factors for UHFLM, UTCLM, and USFLM patients. There was no difference between segmental colon resection and larger colon resection on CSS of UHFLM, UTCLM, and USFLM patients.

**Conclusions:**

We confirmed the different survival of patients with UTCLM, UHFLM, and USFLM, and for the first time, we proved that PTR could provide survival benefits for patients with unresectable CRLM from the perspective of colonic subsites of transverse colon, hepatic flexure, and splenic flexure. Besides, PTR may not improve the prognosis of USFLM patients with CEA- negative or tumor size≤5 cm. For oncologic outcomes, we concluded that segmental colon resection seemed an effective surgical procedure for UTCLM, UHFLM, and USFLM.

## Introduction

Colorectal cancer (CRC) is one of the most common cancers with the second-highest morbidity in men and women and the second leading cause of cancer-related death worldwide. The morbidity of CRC has increased continuously in recent years, with more than 1.8 million confirmed cases reported in 2018 [1]. Unfortunately, about 30–40% of CRC patients are diagnosed with metastatic CRC, and another 30% will develop metastatic CRC later [2]. Among them, the liver is the most common metastatic site [3–5], and liver metastasis is an important cause of death in patients with CRC [6].

The ideal surgical treatment for patients with colorectal cancer liver metastasis (CRLM) seems to be complete surgical resection of liver metastases at the time of primary tumor resection (PTR). However, patients with smaller or fewer liver metastases and right-sided CRC selected for complete surgical resection are easier to approach operatively [7–9]. At the same time, higher morbidity and mortality associated with complete surgical resection is one of the main reasons to limit its application, so many surgeons recommend the traditional staged approach that includes PTR, followed by systemic chemotherapy then resection of liver metastases for patients without progression of the disease [9–11]. However, at the time of diagnosis, 75–90% of CRC patients are unable to undergo surgical resection because of liver metastasis [12]. For these patients with unresectable CRLM, the guidelines of the National Comprehensive Cancer Network (NCCN) do not recommend PTR unless there is obstruction, acute bleeding, or perforation [13]. However, growing evidence has shown that PTR could prolong the survival of patients with unresectable CRLM [14–17].

However, as a junctional site between the right and left colon, lymphatic drainage and vascular supply of the transverse colon including flexures lie between the right and left anatomical territories and their anatomopathological features have not been fully elucidated. Because of this complexity, it seems that this colon segment can not be simply classified as the right colon or the left colon [18–20], and liver metastasis from cancer of this colon segment is more complex than other colon segments. Therefore, it was necessary to conduct targeted research for unresectable CRLM of this colon segment. The purpose of this study was to use the SEER database to evaluate the effect of PTR on the prognosis of patients with unresectable transverse colon cancer liver metastasis (UTCLM), unresectable hepatic flexure cancer liver metastasis (UHFLM), and unresectable splenic flexure cancer liver metastasis (USFLM).

## Patients and methods

### Data source and selection

The SEER 18 regions database [Incidence-SEER 18 Regs Research Data (with additional treatment fields), Nov 2018 Sub (1975–2016 varying)] was used to identify patients with unresectable carcinomas of the transverse colon including flexures with liver metastasis. The selection criteria included: 1) ICD-O-3 site codes: hepatic flexure, transverse colon, and splenic flexure; 2) ICD-O-3 behavior codes: malignant; 3) diagnostic confirmation: positive histology; 4) ICD-O-3 histology codes: adenocarcinoma (8140–8147, 8210–8211, 8220–8221, and 8260–8263), mucinous adenocarcinoma (8480–8481), and signet ring cell carcinoma (8490); 5) complete information of surgery of primary site; 6)vital status: alive, dead. The exclusion criteria were in the following: 1) incomplete information of surgery of primary site; 2) the code of surgery of primary site:26,27,28,29; 3) with not first tumor; 4) without a histological diagnosis; 5) other metastases site except for liver metastasis; 6)surgery of metastatic sites performed; 7) survival months: unknown.

Refer to the published literature [21, 22], we considered patients who did not have resection of liver metastases as unresectable CRLM. All patients were divided into three major cohorts: UHFLM, UTCLM, and USFLM cohorts. Then all patients in every cohort were divided into two groups according to whether they received PTR. According to the scope of colectomy, patients undergoing PTR were divided into segmental colon resection (SCR) and larger colon resection (LCR) subgroups. The data of the SEER database were publicly available, so this study did not require the approval of the ethics review committee. All the authors signed the research agreement form and got permission to access the SEER database.

### Statistical analysis

The X-tile software (version 3.6.1; Yale University, USA) was used to stratify diagnosis ages, year of diagnosis, and median household income (in tens) of the patients. Overall survival (OS) and cause-specific survival (CSS) were used as the main endpoints. OS was defined as the time from diagnosis to death from every cause, and CSS was defined as the date from the first diagnosis to death caused by this kind of disease. OS and CSS were estimated using the Kaplan-Meier analysis with the log-rank test. Cox proportional hazards regression models were subsequently fitted to evaluate factors independently associated with death. The proportional hazards assumptions were confirmed with log-minus-log survival plots. Risk ratio (HR) and 95% confidence interval (CI) were determined by the Cox proportional hazards regression model and subgroup analysis was performed by forest plot to compare the survival of the patients. SPSS22.0 (IBM, Chicago, Illinois, USA) software was used for data analyses. Statistical significance was set at *P* < 0.05, and all tests were 2-sided. Graph Pad Prism 8 was used to generate the Kaplan-Meier survival curve and forest plots.

## Results

### Baseline characteristics of the patients

**Fig. 1:**
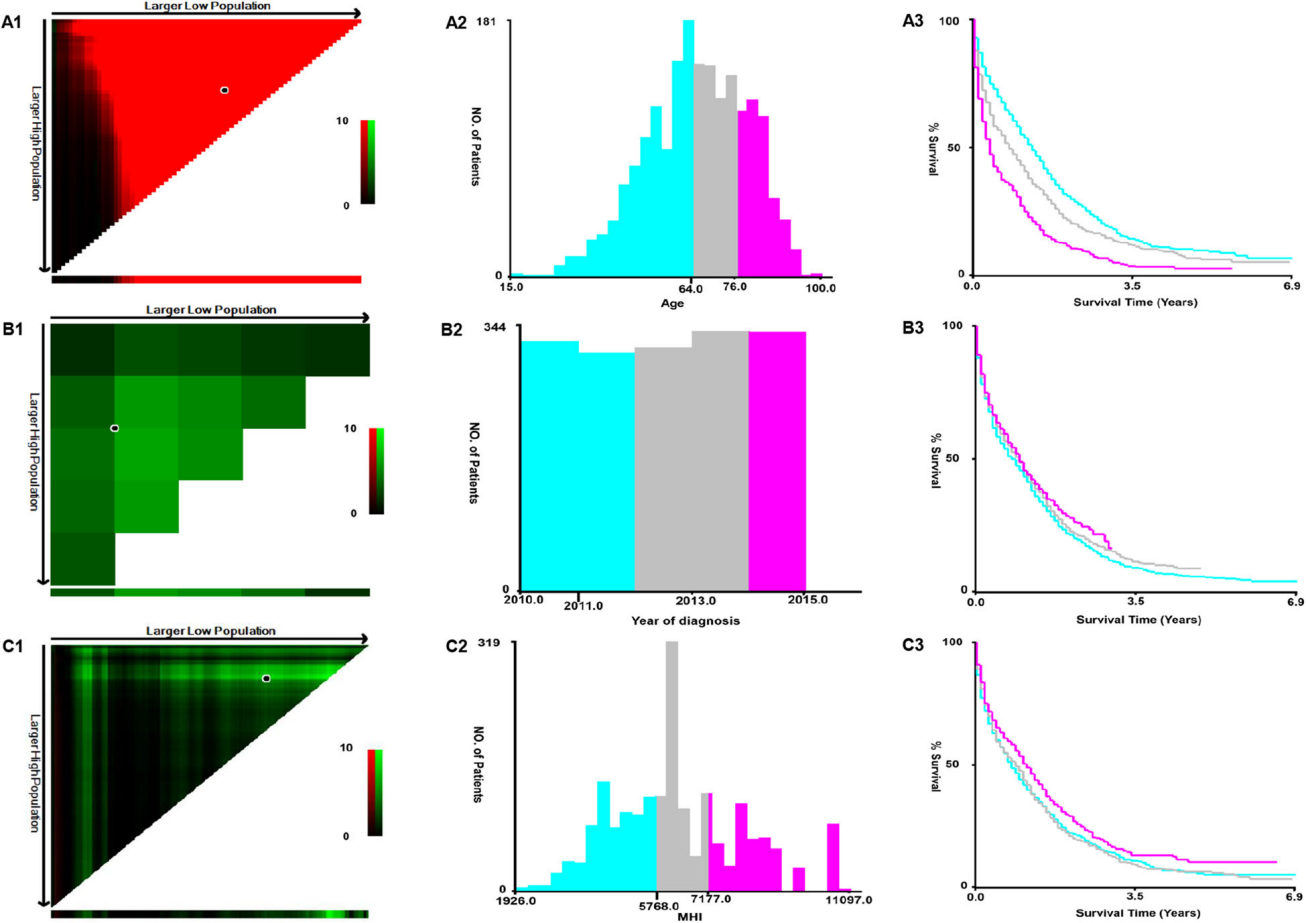
Division of the patients by the optimal cutoff points of age (A1–3), year of diagnosis (B1–3) and median household income (C1–3) produced by the X-tile software. The optimal cutoff points highlighted by the black circle (A1, B1 and C1) are shown on histograms (A2, B2 and C2) and Kaplan-Meier plots (A3, B3 and C3)

Depending on the inclusion criteria, this study included a cohort of 1960 unresectable CRLM patients: 556 cases of UHFLM,1008 cases of UTCLM, and 396 cases of USFLM. The median age of UHFLM, UTCLM, and USFLM cohorts was 66.21 (range 15–100), 65.63 (range 27–93), and 64.12 (range 20–99) years, respectively. There were 227 cases of UHFLM, 563 cases of UTCLM, and 238 cases of USFLM undergoing PTR. Using the X-tile software, cutoff points of age, year of diagnosis, and median household income were yielded (Fig. 1). Table 1 summarized the baseline characteristics of the patients.

**Table 1:** Baseline characteristics of UHFLM, UTCLM and USFLM patients

Variables	Total	UHFLM	UTCLM	USFLM
	1960	556	1008	396
Age				
≤64	920	249(44.8)	458(45.4)	213(53.8)
65–76	567	167(30.0)	301(29.9)	99(25.0)
≥77	473	140(25.2)	249(24.7)	84(21.2)
Gender				
Female	882	242(43.5)	458(45.4)	182(46.0)
Male	1078	314(56.5)	550(54.6)	214(54.0)
Race				
White	1416	406(73.0)	729(72.3)	281(71.0)
Black	376	100(18.0)	200(19.8)	76(19.2)
Other	168	50(9.0)	79(7.8)	39(9.8)
Year of diagnosis				
2010–2011	631	183(32.9)	311(30.9)	137(34.6)
2012–2013	650	176(31.7)	345(34.2)	129(32.6)
2014–2015	679	197(35.4)	352(34.9)	130(32.8)
Marital status				
Unmarried	968	265(477)	516(51.2)	187(47.2)
Married	992	291(523)	492(48.8)	209(52.8)
MHI (in tens)				
1926–5768	767	209(37.6)	402(39.9)	156(39.4)
5769–7177	650	196(35.3)	325(32.2)	129(32.6)
7178–11,097	543	151(27.2)	281(27.9)	111(28.0)
Grade				
I + II	1049	272(48.9)	551(54.7)	226(57.1)
III + IV	492	151(27.2)	253(25.1)	88(22.2)
Unknown	419	133(23.9)	204(20.2)	82(20.7)
T stage				
T1 + T2 + T3	866	212(38.1)	479(47.5)	175(44.2)
T4	554	143(25.7)	278(27.6)	133(33.6)
Unknown	540	201(36.2)	251(24.9)	88(22.2)
N stage				
N0	628	179(32.2)	321(31.8)	128(32.3)
N1 + N2	1103	295(53.1)	584(57.9)	224(56.6)
Unknown	229	82(14.7)	103(10.2)	44(11.1)
Tumor size				
≤5 cm	671	175(31.5)	360(35.7)	136(34.3)
> 5 cm	691	167(30.0)	374(37.1)	150(37.9)
Unknown	598	214(38.5)	274(27.2)	110(27.8)
CEA				
Negative	196	52(9.4)	115(11.4)	29(7.3)
Positive	1204	352(63.3)	609(60.4)	243(61.4)
Unknown	560	152(27.3)	284(28.2)	124(31.3)
PTR				
No	932	329(59.2)	445(44.1)	158(39.9)
Yes	1028	227(40.8)	563(55.9)	238(60.1)
Chemotherapy				
No	763	223(40.1)	396(39.3)	144(36.4)
Yes	1197	333(59.9)	612(60.7)	252(63.6)

*Abbreviations*: *MHI* median household income, *UHFLM* unresectable hepatic flexure cancer liver metastasis, *UTCLM* unresectable transverse colon cancer liver metastasis, *USFLM* unresectable splenic flexure cancer liver metastasis, *CEA* carcinoembryonic antigen; PTR, primary tumor resection

### Kaplan-Meier survival analysis

Of all the 1960 patients finally recruited, 1637 (486 UHFLM, 848 UTCLM, and 303 USFLM) patients had died by the end of the last follow-up, 1540 (459 UHFLM, 793 UTCLM, and 288 USFLM) of whom died of UHFLM, UTCLM, and USFLM specifically. The median survival time of all patients was 11.0 months, ranging from 7.0 months (95% CI 5.404–8.596 months) for UHFLM patients to 15.0 months (95% CI 12.567–17.433 months) for USFLM patients.

**Fig. 2:**
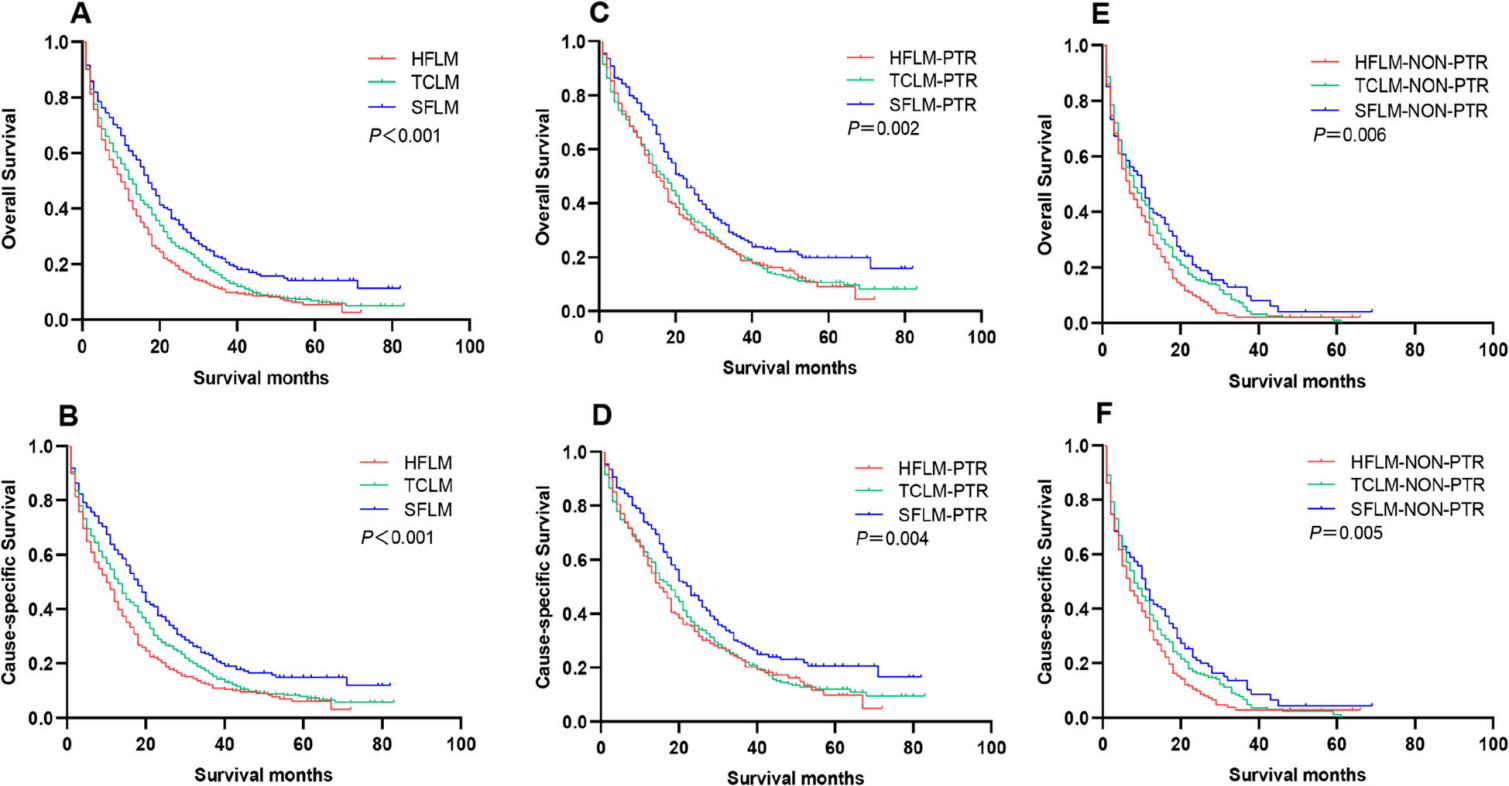
Kaplan-Meier survival analysis for OS and CSS of total cohort (**a** and **b**), PTR cohort (**c** and **d**) and non-PTR cohort (**e** and **f**). Abbreviation: PTR, primary tumor resection; OS, overall survival; CSS, cause-specific survival

The results of the survival analysis of all patients were shown in Fig. 2 and Table 2. USFLM patients had the best OS, with 1-year OS rate of 60.8%, 3-year OS rate of 22.1%, and 5-year OS rate of 14.2%, followed by UTCLM patients (with 1-year OS rate of 50.6%, 3-year OS rate of 15.1%, and 5-year OS rate of 6.9%, respectively). UHFLM patients had the worst OS: the 1-year OS rate of 42.8%, 3-year OS rate of 11.0%, and 5-year OS rate of 5.5%, respectively (*P* < 0.001). Similarly, USFLM patients had the best CSS, with 1-year CSS rate of 61.7%, 3-year CSS rate of 23.0%, and 5-year CSS rate of 14.9%, followed by UTCLM patients (with 1-year CSS rate of 51.2%, 3-year CSS rate of 16.3%, and 5-year CSS rate of 7.8% respectively). UHFLM patients had the worst CSS: the 1-year CSS rate of 42.8%, 3-year CSS rate of 12.0%, and 5-year CSS rate of 6.0%, respectively (*P* < 0.001).

**Table 2:** Survival analysis for OS and CSS of UHFLM, UTCLM and USFLM patients

		OS			CSS		
		1 year(%)	3 years(%)	5 years(%)	1 year(%)	3 years(%)	5 years(%)
UHFLM	Total	42.8	11.0	5.5	42.8	12.0	6.0
	Non-PTR	31.6	2.1	2.1	31.8	2.8	2.8
	PTR	57.8	21.2	9.1	57.7	22.6	9.7
UTCLM	Total	50.6	15.1	6.9	51.2	16.3	7.8
	Non-PTR	37.5	5.8	1.1	37.8	6.5	1.2
	PTR	59.7	21.2	10.7	60.5	22.7	12.0
USFLM	Total	60.8	22.1	14.2	61.7	23.0	14.9
	Non-PTR	41.2	13.0	4.1	43.2	13.7	4.3
	PTR	72.9	27.7	19.8	72.9	28.8	20.6

*Abbreviations*: *OS* overall survival, *CSS* cause-specific survival, *PTR* primary tumor resection, *UHFLM* unresectable hepatic flexure cancer liver metastasis, *UTCLM* unresectable transverse colon cancer liver metastasis, *USFLM* unresectable splenic flexure cancer liver metastasis

### Prognostic factors

Univariate and multivariate Cox regression analyses for OS of UHFLM, UTCLM, and USFLM patients were performed (Table 3). The common independent prognostic factors for UHFLM, UTCLM, and USFLM patients included age (≤64 vs. ≥77), grade (I + II vs. III + IV), PTR (no vs. yes), and chemotherapy (no vs. yes). N stage (N0 vs. N1 + N2) were independent prognostic factors for UHFLM and UTCLM patients but not for USFLM patients; T stage (T1 + T2 + T3 vs. T4) was an independent prognostic factor for UHFLM and USFLM patients but not for UTCLM patients; year of diagnosis (2010–2011 vs. 2014–2015) was an independent prognostic factor for UTCLM and USFLM patients but not for UHFLM patients; CEA (negative vs. positive) was only an independent prognostic factor for UTCLM patients but not for UHFLM and USFLM patients.

**Table 3:** Univariate and multivariate analysis for OS of UHFLM, UTCLM and USFLM patients

Variables	UHFLM				UTCLM				USFLM			
	Univariate analysis		Multivariate analysis		Univariate analysis		Multivariate analysis		Univariate analysis		Multivariate analysis	
Age	HR (95%CI)	*P* value	HR (95%CI)	*P* value	HR (95%CI)	*P* value	HR (95%CI)	*P* value	HR (95%CI)	*P* value	HR (95%CI)	*P* value
≤64	Reference		Reference		Reference		Reference		Reference		Reference	
65–76	1.397 (1.132–1.726)	0.002	1.278 (1.023–1.595)	0.030	1.245 (1.060–1.462)	0.008	1.273 (1.078–1.503)	0.004	1.193 (0.902–1.579)	0.215	1.162 (0.866–1.559)	0.316
≥77	1.737 (1.393–2.167)	<0.001	1.407 (1.102–1.795)	0.006	1.799 (1.522–2.126)	<0.001	1.482 (1.234–1.779)	<0.001	2.784 (2.110–3.673)	<0.001	1.924 (1.400–2.643)	<0.001
Gender												
Female	Reference		Reference		Reference		Reference		Reference		Reference	
Male	1.004 (0.839–1.201)	0.969	0.987 (0.816–1.193)	0.889	1.014 (0.886–1.161)	0.848	1.049 (0.913–1.205)	0.501	1.103 (0.879–1.385)	0.396	1.122 (0.879–1.432)	0.357
Race												
White	Reference		Reference		Reference		Reference		Reference		Reference	
Black	1.084 (0.859–1.368)	0.499	1.185 (0.927–1.514)	0.176	0.938 (0.792–1.111)	0.458	0.995 (0.832–1.191)	0.959	1.118 (0.844–1.482)	0.437	1.288 (0.945–1.756)	0.110
Other	0.900 (0.656–1.235)	0.514	0.823 (0.582–1.164)	0.271	0.869 (0.674–1.121)	0.280	0.869 (0.671–1.127)	0.291	0.838 (0.565–1.244)	0.381	0.870 (0.572–1.323)	0.514
Year of diagnosis												
2010–2011	Reference		Reference		Reference		Reference		Reference		Reference	
2012–2013	0.822(0.662–1.021)	0.076	0.850 (0.682–1.061)	0.151	0.921 (0.785–1.081)	0.313	0.816 (0.691–0.963)	0.016	0.948 (0.731–1.230)	0.688	0.857 (0.848–1.135)	0.282
2014–2015	0.892(0.716–1.112)	0.311	0.813 (0.648–1.019)	0.073	0.793 (0.667–0.944)	0.009	0.778 (0.650–0.931)	0.006	0.851 (0.634–1.142)	0.282	0.647 (0.474–0.883)	0.006
Marital status												
Unmarried	Reference		Reference		Reference		Reference		Reference		Reference	
Married	0.878 (0.734–1.049)	0.151	1.032 (0.854–1.248)	0.743	0.853 (0.745–0.976)	0.020	0.876 (0.760–1.010)	0.069	0.763 (0.609–0.957)	0.019	0.899 (0.706–1.144)	0.386
MHI (in tens)												
1926–5768	Reference		Reference		Reference		Reference		Reference		Reference	
5769–7177	0.958 (0.780–1.177)	0.685	0.933 (0.753–1.156)	0.526	1.073 (0.717–1.255)	0.381	1.047 (0.890–1.232)	0.579	0.858 (0.657–1.119)	0.258	1.132 (0.854–1.500)	0.390
7178–11,097	0.763 (0.607–0.958)	0.020	0.735 (0.574–1.940)	0.014	0.914 (0.771–1.082)	0.295	0.952 (0.796–1.137)	0.586	0.732 (0.554–0.968)	0.029	0.935 (0.689–1.269)	0.665
Grade												
I + II	Reference		Reference		Reference		Reference		Reference		Reference	
III + IV	1.275 (1.029–1.579)	0.026	1.555 (1.242–1.945)	<0.001	1.526 (1.298–1.794)	<0.001	1.651 (1.392–1.957)	<0.001	1.387 (1.049–1.833)	0.022	1.375 (1.024–1.846)	0.034
Unknown	1.544 (1.236–1.928)	<0.001	1.082 (0.850–1.378)	0.520	1.813 (1.523–2.158)	<0.001	1.373 (1.129–1.671)	0.001	1.687 (1.271–2.239)	<0.001	0.998 (0.702–1.417)	0.990
T stage												
T1 + T2 + T3	Reference		Reference		Reference		Reference		Reference		Reference	
T4	1.413 (1.123–1.779)	0.003	1.511 (1.194–1.913)	0.001	1.266 (1.077–1.487)	0.004	1.113 (0.941–1.317)	0.210	1.220 (0.939–1.586)	0.136	1.444 (1.086–1.920)	0.011
Unknown	1.773 (1.435–2.191)	<0.001	1.213 (0.931–1.581)	0.152	1.689 (1.430–1.994)	<0.001	0.997 (0.805–1.235)	0.977	1.831 (1.373–2.442)	<0.001	1.322 (0.904–1.933)	0.151
N stage												
N0	Reference		Reference		Reference		Reference		Reference		Reference	
N1 + N2	0.990 (0.809–1.210)	0.918	1.489 (1.192–1.861)	<0.001	0.942 (0.810–1.095)	0.437	1.463 (1.231–1.739)	<0.001	0.801 (0.625–1.027)	0.080	1.177 (0.879–1.577)	0.274
Unknown	1.603 (1.218–2.109)	<0.001	1.400 (1.042–1.882)	0.026	1.913 (1.516–2.413)	<0.001	1.418 (1.102–1.835)	0.007	1.399 (0.966–2.026)	0.076	0.876 (0.576–1.332)	0.535
Tumor size												
≤5 cm	Reference		Reference		Reference		Reference		Reference		Reference	
> 5 cm	1.199 (0.951–1.511)	0.125	0.968 (0.761–1.232)	0.794	1.247 (1.062–1.464)	0.007	1.075 (0.910–1.270)	0.393	1.435 (1.093–1.884)	0.009	1.170 (0.877–1.561)	0.287
Unknown	1.668 (1.340–2.077)	<0.001	0.928 (0.707–1.219)	0.593	1.832 (1.543–2.176)	<0.001	1.192 (0.970–1.466)	0.095	1.830 (1.366–2.451)	<0.001	0.932 (0.633–1.372)	0.721
CEA												
Negative	Reference		Reference		Reference		Reference		Reference		Reference	
Positive	1.373 (0.984–1.917)	0.062	1.135 (0.807–1.595)	0.467	1.562 (1.237–1.973)	<0.001	1.422 (1.118–1.807)	0.004	1.680 (1.046–2.698)	0.032	1.360 (0.830–2.226)	0.222
Unknown	1.714 (1.200–2.448)	0.003	1.096 (0.757–1.586)	0.627	1.623 (1.263–2.086)	<0.001	1.384 (1.073–1.787)	0.012	1.804 (1.101–2.955)	0.019	1.366 (0.812–2.299)	0.240
PTR												
No	Reference		Reference		Reference		Reference		Reference		Reference	
Yes	0.487 (0.402–0.589)	<0.001	0.341 (0.257–0.453)	<0.001	0.539 (0.470–0.619)	<0.001	0.431 (0.345–0.538)	<0.001	0.479 (0.380–0.603)	<0.001	0.390 (0.267–0.570)	<0.001
Chemotherapy												
No	Reference		Reference		Reference		Reference		Reference		Reference	
Yes	0.390(0.324–0.469)	<0.001	0.343 (0.276–0.425)	<0.001	0.334 (0.290–0.384)	<0.001	0.326 (0.280–0.380)	<0.001	0.285 (0.225–0.361)	<0.001	0.284 (0.214–0.377)	<0.001

*Abbreviations*: *OS* overall survival, *UHFLM* unresectable hepatic flexure cancer liver metastasis, *UTCLM* unresectable transverse colon cancer liver metastasis, *USFLM* unresectable splenic flexure cancer liver metastasis, *MHI* median household income, *PTR* primary tumor resection

### Survival analysis for OS and CSS between the PTR and non-PTR groups

**Fig. 3:**
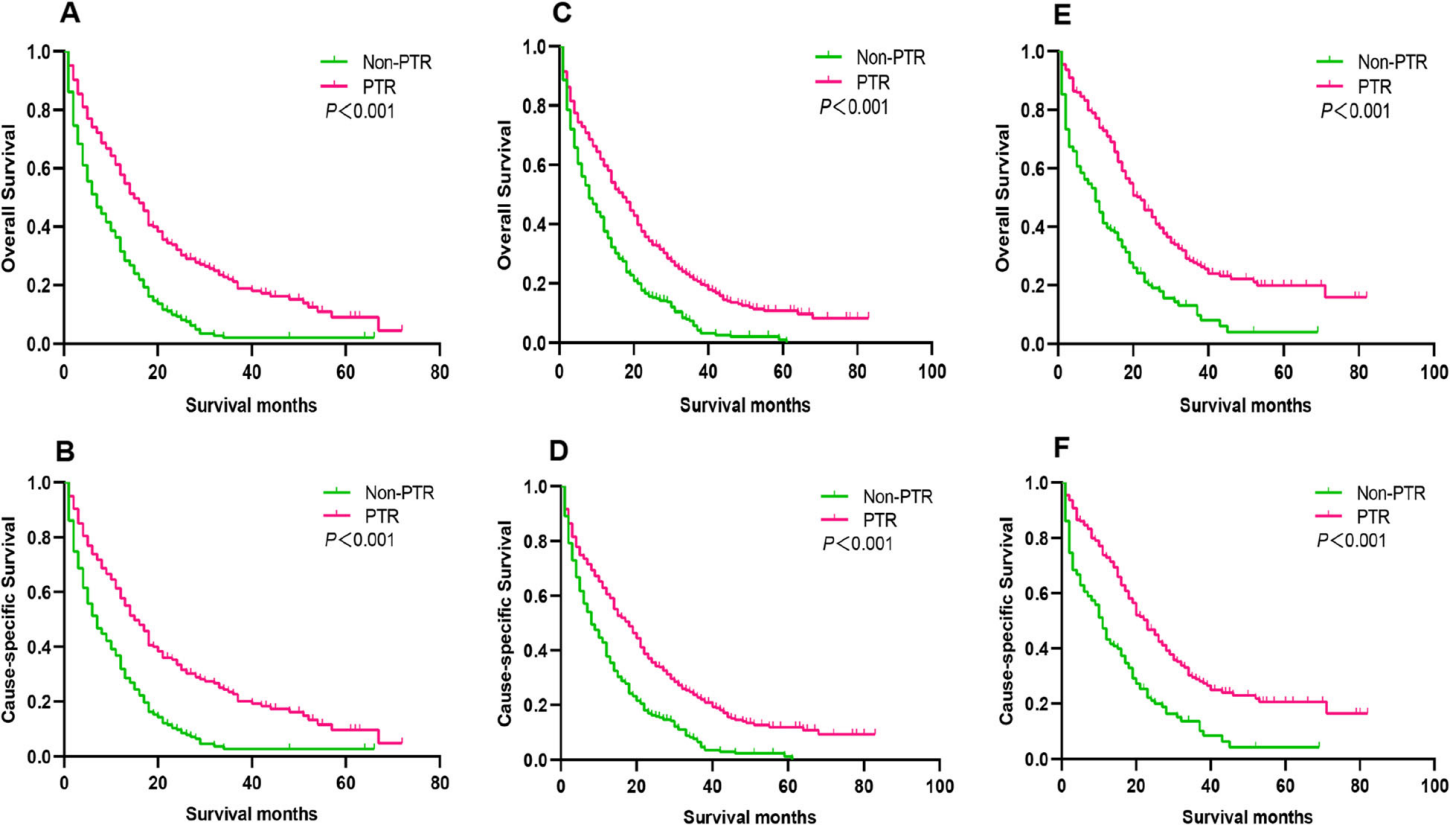
Kaplan-Meier Survival analysis for OS and CSS between the PTR and non-PTR groups in UHFLM (**a** and **b**), UTCLM (**c** and **d**) and USFLM (**e** and **f**) patients. Abbreviation: PTR, primary tumor resection; OS, overall survival; CSS, cause-specific survival; UHFLM, unresectable hepatic flexure cancer liver metastasis; UTCLM, unresectable transverse colon cancer liver metastasis; USFLM, unresectable splenic flexure cancer liver metastasis

For UHFLM patients, the 1-year, 3-year and 5-year OS rate of PTR vs. non-PTR groups were 57.8% vs. 31.6, 21.2% vs. 2.1 and 9.1% vs. 2.1%, respectively (*P*<0.001). The 1-year, 3-year and 5-year CSS rate of PTR vs. non-PTR groups were 57.7% vs. 31.8, 22.6% vs. 2.8 and 9.7% vs. 2.8%, respectively (*P*<0.001) (Fig. 3 and Table 2).

For UTCLM patients, the 1-year, 3-year and 5-year OS rate of PTR vs. non-PTR groups were 59.7% vs. 37.5, 21.2% vs. 5.8 and 10.7% vs. 1.1%, respectively (*P*<0.001). The 1-year, 3-year and 5-year CSS rate of PTR vs. non-PTR groups were 60.5% vs. 37.8, 22.7% vs. 6.5 and 12.0% vs. 1.2%, respectively (*P*<0.001) (Fig. 3 and Table 2).

For USFLM patients, the 1-year, 3-year and 5-year OS rate of PTR vs. non-PTR groups were 72.9% vs. 41.2, 27.7% vs. 13.0 and 19.8% vs. 4.1%, respectively (*P*<0.001). The 1-year, 3-year and 5-year CSS rate of PTR vs. non-PTR groups were 72.9% vs. 43.2, 28.8% vs. 13.7 and 20.6% vs. 4.3%, respectively (*P*<0.001) (Fig. 3 and Table 2).

### Subgroup analyses for OS and CSS

Subgroup analyses for OS and CSS were performed in prespecified subgroups using forest plots. The prespecified stratification factor was whether PTR was performed.

**Fig. 4:**
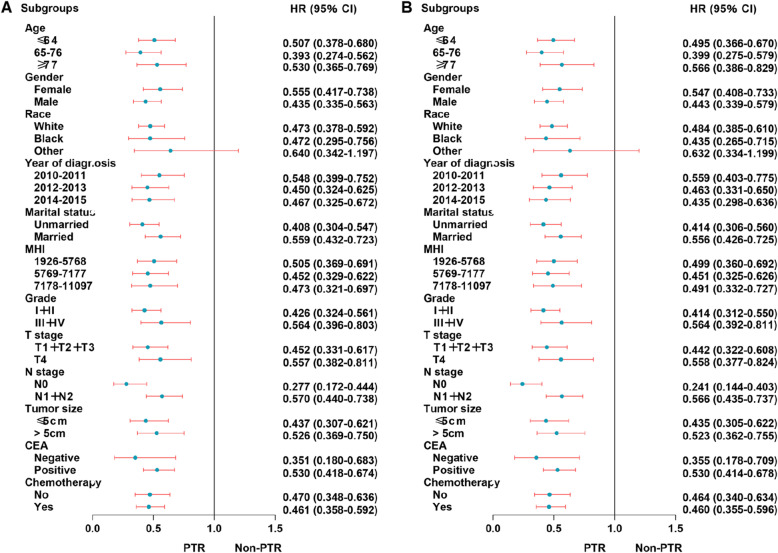
Forest plot for UHFLM patients in the subgroup analysis of OS (**a**) and CSS (**b**). Abbreviation: UHFLM, unresectable hepatic flexure cancer liver metastasis; MHI, median household income

For the UHFLM group, the forest plot showed that there were no statistical differences in the patients of other race (HR 0.640; 95% CI 0.342–1.197) subgroup for OS when the efficacy of PTR to non-PTR was compared; there were no statistical differences in the patients with other race (HR 0.632; 95% CI 0.334–1.199) subgroup for CSS. Other subgroups showed significant statistical differences for OS and CSS (Fig. 4).

**Fig. 5:**
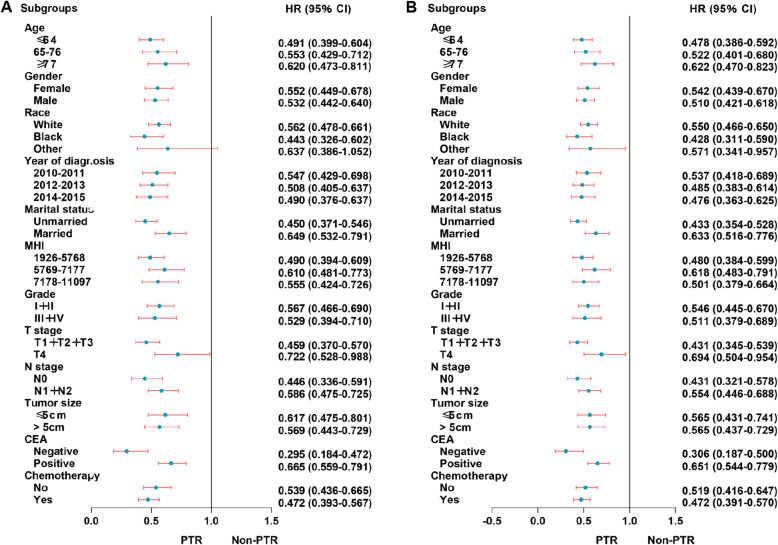
Forest plot for UTCLM patients in the subgroup analysis of OS (**a**) and CSS (**b**). Abbreviation: UTCLM, unresectable transverse colon cancer liver metastasis; MHI, median household income

For the UTCLM group, the forest plot showed that there were no statistical differences in the patients of other race (HR 0.637; 95% CI 0.386–1.052) subgroup for OS when the efficacy of PTR to non-PTR was compared. Other subgroups showed significant statistical differences for OS and CSS (Fig. 5).

**Fig. 6:**
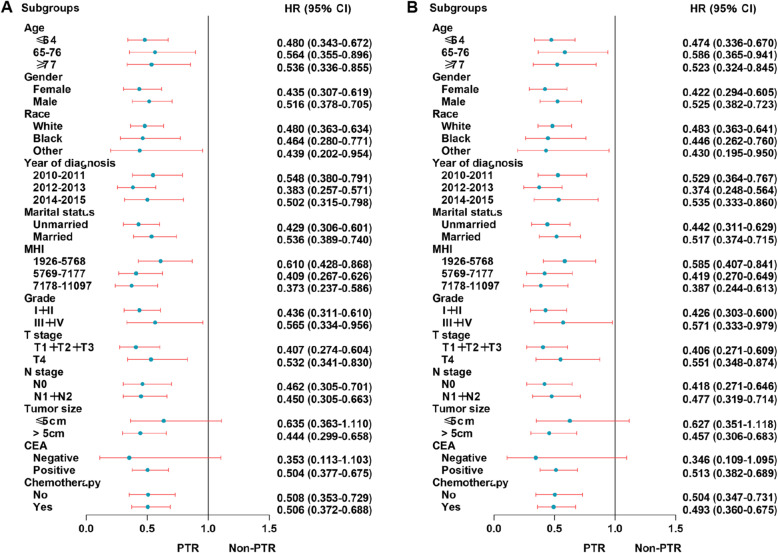
Forest plot for USFLM patients in the subgroup analysis of OS (**a**) and CSS (**b**). Abbreviation: USFLM, unresectable splenic flexure cancer liver metastasis; MHI, median household income

For the USFLM group, the forest plot showed that there were no statistical differences in the patients with tumor size≤5 cm (HR 0.635; 95% CI 0.363–1.110) and negative CEA(HR 0.353; 95% CI 0.113–1.103) subgroups for OS when the efficacy of PTR to non-PTR was compared; there were no statistical differences in the patients with tumor size≤5 cm (HR 0.627; 95% CI 0.351–1.118) and negative CEA (HR 0.346; 95% CI 0.109–1.095) subgroups for CSS. Other subgroups showed significant statistical differences for OS and CSS (Fig. 6).

### Survival analysis for OS and CSS between the SCR and LCR groups according to the scope of colectomy

**Fig. 7:**
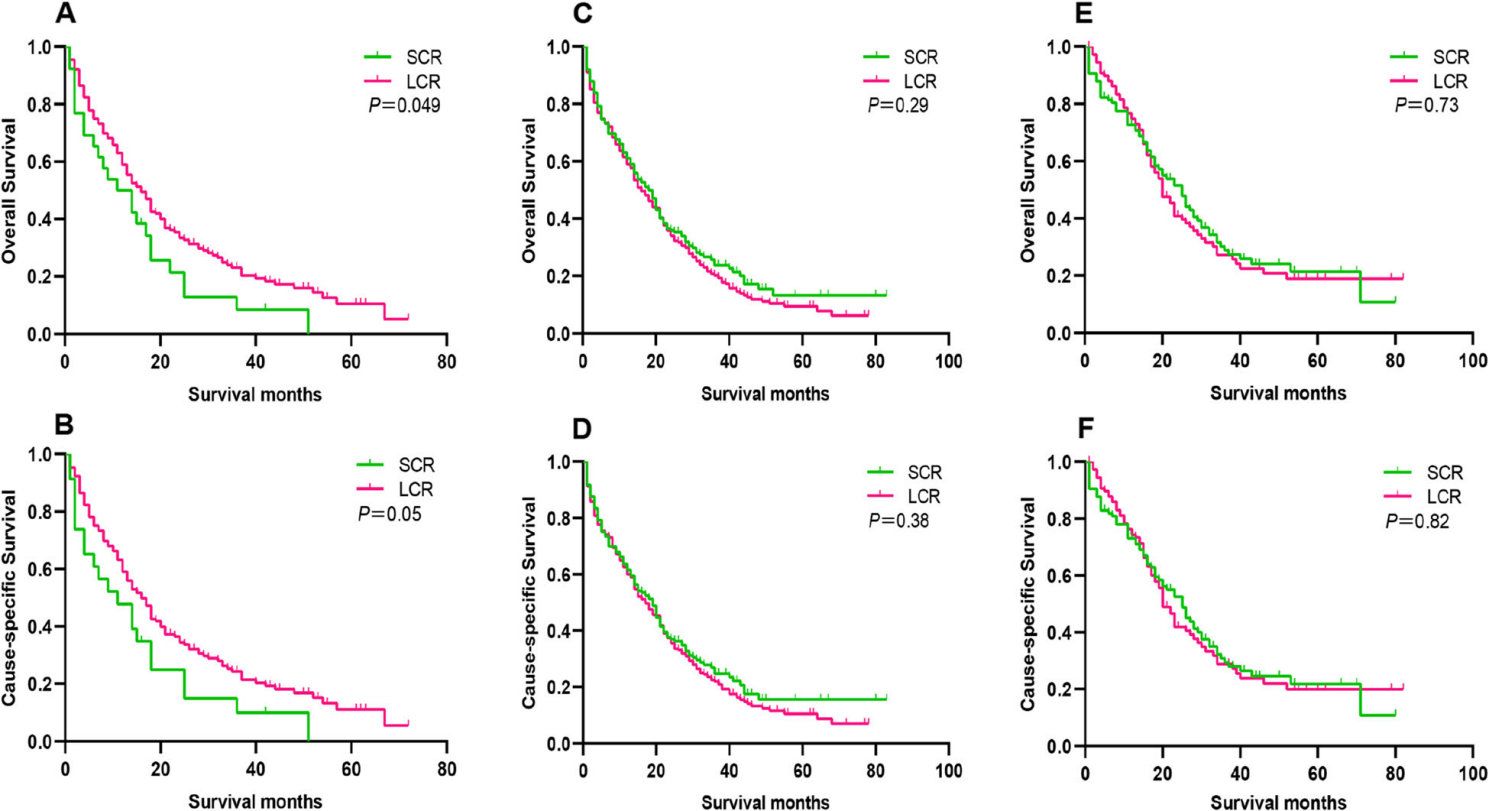
Kaplan-Meier Survival analysis for OS and CSS between the SCR and LCR groups in UHFLM (**a** and **b**), UTCLM (**c** and **d**) and USFLM (**e** and **f**) patients. Abbreviation: SCR, segmental colon resection; LCR, larger colon resection; OS, overall survival; CSS, cause-specific survival; UHFLM, unresectable hepatic flexure cancer liver metastasis; UTCLM, unresectable transverse colon cancer liver metastasis; USFLM, unresectable splenic flexure cancer liver metastasis

According to the scope of colectomy, UHFLM, UTCLM and USFLM patients undergoing PTR were further divided into SCR and LCR subgroups. For UHFLM patients, the 1-year, 3-year and 5-year OS rate of SCR vs. LCR groups were 50.0% vs. 59.0, 8.5% vs. 23.1 and 0.0% vs. 10.5%, respectively (*P* = 0.049); the 1-year, 3-year and 5-year CSS rate of SCR vs. LCR groups were 47.8% vs. 59.0, 9.9% vs. 24.3 and 0.0% vs. 11.0%, respectively (*P* = 0.05). For UTCLM patients, the 1-year, 3-year and 5-year OS rate of SCR vs. LCR groups were 61.1% vs. 59.1, 23.7% vs. 20.2 and 13.2% vs. 9.5%, respectively (*P* = 0.29); the 1-year, 3-year and 5-year CSS rate of SCR vs. LCR groups were 61.7% vs. 60.0, 24.7% vs. 22.0 and 15.5% vs. 10.6%, respectively (*P* = 0.38). For USFLM patients, the 1-year, 3-year and 5-year OS rate of SCR vs. LCR groups were 72.7% vs. 74.9, 28.8% vs. 27.2 and 21.4% vs. 18.9%, respectively (*P* = 0.73); the 1-year, 3-year and 5-year CSS rate of SCR vs. LCR groups were 73.2% vs. 74.4, 29.4% vs. 28.8 and 21.8% vs. 20.0%, respectively (*P* = 0.82) (Fig. 7 and Table 4).

**Table 4:** Survival analysis for OS and CSS between the SCR and LCR groups

		OS			CSS		
		1 year(%)	3 years(%)	5 years(%)	1 year(%)	3 years(%)	5 years(%)
UHFLM	SCR	50.0	8.5	0.0	47.8	9.9	0.0
	LCR	59.0	23.1	10.5	59.0	24.3	11.0
UTCLM	SCR	61.1	23.7	13.2	61.7	24.7	15.5
	LCR	59.1	20.2	9.5	60.0	22.0	10.6
USFLM	SCR	72.7	28.8	21.4	73.2	29.4	21.8
	LCR	74.9	27.2	18.9	74.4	28.8	20.0

*Abbreviations*: *OS* overall survival, *CSS* cause-specific survival, *PTR* primary tumor resection, *SCR* segmental colon resection, *LCR* larger colon resection, *UHFLM* unresectable hepatic flexure cancer liver metastasis, *UTCLM* unresectable transverse colon cancer liver metastasis, *USFLM* unresectable splenic flexure cancer liver metastasis

## Discussion

Previously, the effect of tumor location on the prognosis of patients with unresectable metastatic CRC was compared between two or three groups mostly according to “right colon and left colon” and “right colon, left colon and rectum” [22–26]. However, more and more studies proved that a simple classification into right- and left-sided CRC could not represent the complexity of this tumor entity, and put forward the importance of researching from the perspective of colonic subsites [27–29]. As a continuum from right to the left colon, complex blood supply, and lymphatic drainage caused transverse colon including flexures the most complex colon segment in the whole colon, but there were few studies on liver metastasis from cancer of this colon segment. As far as we know, this was the first time to study the prognosis of patients with unresectable CRLM and the effect of PTR on their survival from colonic subsites of transverse colon, hepatic flexure, and splenic flexure. We found that for the total cohort, the survival of UHFLM was poorer than that of UTCLM and USFLM. For patients undergoing PTR, there was no difference in prognosis between UHFLM and UTCLM, but the prognosis of USFLM was significantly better than that of UHFLM and UTCLM. For non-PTR patients, the prognosis of UHFLM was poorer than that of UTCLM and USFLM, but there was no difference between UTCLM and USFLM. In short, regardless of whether the patients with UHFLM, UTCLM, and USFLM undergoing PTR, the prognosis of UHFLM were poorer. In response to these results, we proposed possible explanations. Firstly, different embryonic sources of these three colonic subsites: in the embryologic development of the distal intestine, hepatic flexure originates from the midgut, and splenic flexure originates from the hindgut. One study found that embryonic origin was involved in the prognosis of metastatic CRC [30], and a subsequent study demonstrated that 5-year OS in patients with hindgut-derived CRC was better than that in patients with midgut-derived CRC [31]. This may explain why the prognosis of UHFLM was poorer and that of USFLM was better, while the prognosis of UTCLM was different in different cohorts, which may be due to the complex oncological characteristics of 2/3 of transverse colon originating from the midgut and 1/3 from the hindgut. Another possible explanation was different pathways of liver metastasis from the transverse colon, hepatic flexure, and splenic flexure cancer. The splenic flexure is supplied by the branches of the inferior mesenteric artery and the reflux of the vein mainly flows into the inferior mesenteric vein. The hepatic flexure is mainly supplied by the branches of the superior mesenteric artery, and the reflux of the vein mainly flows into the superior mesenteric vein [32, 33]. According to the theory of “streamline flow of the portal vein”, the blood of the superior mesenteric vein enters the right lobe of the liver along the right side of the portal vein, while the blood of the splenic vein enters the left lobe of the liver along the left side of the portal vein [34]. We speculated hepatic flexure cancer was more metastases to the right lobe and splenic flexure cancer was more metastases to the left lobe of the liver. It was reported that there were more metastases in the right lobe of the liver than in left lobe [35, 36], which may lead to a heavier tumor burden in the right lobe and a worse prognosis. However, more in-depth targeted research is required.

Previous studies have shown that PTR could prolong the survival of patients with unresectable metastatic CRC [23–26]. Different from the previous studies, we analyzed the effect of PTR on the survival of unresectable CRLM from three colonic subsites of the transverse colon, hepatic flexure, and splenic flexure for the first time. We found that PTR was a common and independent factor for UHFLM, UTCLM, and USFLM, and PTR could prolong the OS and CSS of the patients. These results were encouraging because we provided evidence that PTR could prolong the survival of patients with unresectable CRLM of the most complex colonic segment. We speculated that there were several possible reasons why PTR could improve the survival of the patients: first, the increased survival rate after PTR may be attributed not only to the reduction of primary tumor burden, but also to the reduction of cancer stem cells resistant to chemotherapy [37–39]; second, PTR reduced the potential CRC-related complications, such as acute bleeding, perforation, and obstruction, which could cause higher surgical mortality and morbidity [40, 41]; third, PTR may restore the immunosuppressive effect caused by metastatic tumors, which has been confirmed in animal models [42]; forth, based on Stephen Paget’s “seed and soil” theory [43], PTR destroyed the angiogenic environment favoring unresectable liver metastasis growth [44].

In practical clinical work, the acceptance of PTR, in patients with unresectable CRLM was mostly based on the existence of metastatic symptoms. However, studies pointed out that PTR for unresectable metastatic CRC should be based on metastatic tumor burden, not just on the presence of symptoms of metastatic disease [45]. Currently, the most clinical prognostic scoring systems used to evaluate tumor burden regarding the number and size of metastatic lesions as the main index [7, 11, 46]. The tumor burden score (TBS) scoring system widely used [47] and the genetic and morphological evaluation (GAME) scoring system [48] based on the TBS take the maximum diameter of metastatic lesions in pathological specimens as the horizontal axis and the numbers of metastatic lesions as the longitudinal axis to construct a coordinate system to evaluate the tumor burden. The TBS score system has been used to evaluate the burden of liver metastasis to guide surgery for patients with CRLM, especially R0 resection [49, 50]. A recent study assessed the effect of PTR on the prognosis of patients with unresectable metastatic CRC (M1a disease and M1b disease). The authors found that PTR could prolong the survival time of the patients with M1a disease and M1b disease, but patients with M1a disease got more clinical benefits from PTR than patients with M1b disease [14]. These results indicated that patients with unresectable metastatic CRC with two or more metastatic organs had a higher tumor burden than patients with metastasis to one organ, which seemed to be the main reason why they benefit less from PTR. However, they did not further stratify M1b disease, so it was difficult to determine whether the higher tumor burden of M1b disease came from liver combined with other multi-organ metastasis or multi-organ metastasis except liver. Because the liver is the main metastatic target organ of metastatic CRC [3–5], it is necessary to stratify the tumor burden with liver metastasis as the core to further analyze the effect of PTR on the prognosis of patients with unresectable CRLM. The TBS and GAME scoring systems may solve this limitation because the two scoring systems could classify patients more accurately according to the overall tumor burden. However, considering that the patients with CRC included in these scoring systems were only divided into patients from the left colon, right colon, or rectum, patients with unresectable CRLM from different colon subsites such as transverse colon including flexures should be further stratified to determine surgical strategies for the patients (especially unresectable patients). Since this study only included patients with unresectable CRC with simple liver metastasis (M1a disease), and the SEER database did not provide a detailed number and size of metastatic foci of a single organ, which hindered further analysis for the effects of different liver metastatic burden on the survival of patients with unresectable CRLM from transverse colon including flexures.

As a recommended prognostic marker in CRC for tumor diagnosis and monitoring response to therapy, carcinoembryonic antigen (CEA) can protect metastatic cells from death, change the microenvironment of sinusoids, promote the expression of adhesion molecule and malignant cell survival, besides being considered a proangiogenic molecule [51]. Studies showed that CRC patients with elevated CEA levels tend to have a higher incidence of liver metastasis [52, 53], and elevated serum CEA levels in CRC patients were often associated with metastasis after primary resection [54]. Although CEA is considered to promote metastasis and inhibit cell differentiation, there are still CRC patients with normal serum CEA levels with advanced or even recurrent tumors [55, 56]. A recent study showed that lower CEA levels were positively correlated with reduced survival, and CEA-negative CRC cells were more likely to migrate and invade than CEA-positive CRC cells [57]. It could be seen that the role of CEA level in the occurrence and development of metastatic CRC was extremely complex. Our forest plot of subgroup analysis showed that PTR prolonged survival in CEA-positive UTCLM, UHFLM, and USFLM patients and CEA-negative UTCLM and UHFLM patients, but PTR could not provide survival benefits for CEA-negative USFLM patients. This difference has not been reported in previous studies. In this study, the majority of CEA-negative USFLM patients were in T3, T4, N1, and N2 stages. So based on the results of Yan et al. [57], we speculated that CEA-negative USFLM patients had larger primary tumor volume, a wider range of adjacent tissue involvement, and more severe lymph node involvement. This reason seemed to explain the fact that CEA-negative USFLM patients were unable to benefit from PTR.

At present, the scope of primary resection of the transverse colon including flexures cancer mainly includes total colectomy, total proctocolectomy, hemicolectomy, enlarged hemicolectomy, segmental colon resection, and so on [58–62]. In the past, the choice of surgery was more based on the assumption that the larger the resection scope, the more lymph node dissection, and the better the prognosis [63–65]. However, as more studies have evaluated the effects on patient’s prognosis of different resection scopes, compared with LCR, SCR seemed to lead to more satisfactory oncological outcomes [66–73]. However, there was no research on whether SCR could prolong the survival of UTCLM, UHFLM, and USFLM patients. Our results showed that LCR could not lead to a better prognosis than SCR for the patients. In addition to a complete removal of the tumor, adequate lymph node dissection is also important for CRC patients. Due to the complexity of lymphatic drainage of the transverse colon including flexures [74–77], many surgeons preferred enlarged resection and extensive lymph node dissection. Some studies have answered yes to the adequacy of segmental colon dissection, suggesting that SCR could remove the same or less number of lymph nodes than LCR, but there was no difference in prognosis between the two groups [69, 70, 78, 79]. Besides, SCR for transverse colon cancer was associated with less ileus but higher anastomotic leak rates and lower lymph node yields, and similar hospital stay [69]. For splenic flexure cancer, there was no difference in morbidity and mortality, the rate of lymph node yields and survival rate between SCR and LCR groups [68], and the operation time and hospital stay were shorter [71]. However, there was still a lack of reports on SCR for hepatic flexure cancer. In this study, we only initially reported survival outcomes. Due to the lack of surgical data, postoperative complications, recurrence rate, and hospitalization-related information in the SEER database, more comprehensive randomized controlled trials needed to be carried out based on these results.

In addition to surgical resection, perioperative chemotherapy plays a more and more important role in the treatment of unresectable CRLM patients. In order to improve long-term survival by reducing postoperative relapse, and conversion and down-sizing chemotherapy, the main chemotherapy regimen for patients with unresectable CRLM is systemic therapy with oxaliplatin- or irinotecan-based chemotherapy (FOLFOX, FOLFIRI) combined with targeted agents, such as anti-vascular endothelial growth factor (anti-VEGF) bevacizumab, or epidermal growth factor receptor (EGFR) inhibitor cetuximab [80]. Currently, because of higher response and resection rates, the chemotherapy regimen for unresectable CRLM patients is more inclined to triple chemotherapy regimen (FOLFOXIRI) [81, 82]. Our analysis of the prognostic factors of unresectable CRLM patients showed that perioperative chemotherapy was a common independent prognostic factor, and the prognosis of UTCLM, UHFLM, and USFLM patients who received perioperative chemotherapy was significantly better than that of patients without perioperative chemotherapy. This did not seem to be an unexpected result, as many studies have shown the positive effect of perioperative chemotherapy on the prognosis of unresectable CRLM [81–83], especially when mutational status analysis has been used by some guidelines to guide the treatment and prognosis of CRLM [84, 85]. Studies showed that not only KRAS, BRAF could guide the identification of CRLM patients who could benefit most from surgical resection [80, 86]*, but also KRAS, NRAS, BRAF, TP53, MSI, APC, and PIK3CA became important prognostic indexes to guide perioperative chemotherapy in patients with CRLM* [87–91], and could guide the selection of further combined targeted therapy. The latest multicenter phase II study revealed that EGFR inhibitor cetuximab plus modified FOLFOXIRI (5-fluorouracil/folinic acid, oxaliplatin, irinotecan) could significantly improve the rate of no evidence of disease, objective response rate, total survival rate and progression-free survival of BRAF/RAS wild-type unresectable CRLM patients [92], and another study also showed that cetuximab based on systemic chemotherapy could increase the resectable rate and R0 resection rate in patients with KRAS wild-type [93]. However, CRC patients with KRAS [94] and NRAS [95] gene mutations, possibly due to mutations in downstream genes such as BRAF [96], have been demonstrated to be insensitive to treatment with EGFR inhibitor. On the other hand, anti-VEGF bevacizumab combined with FOLFOXIRI could improve median progression-free survival, overall tumor response rates, and R0 resection rates in patients with unresectable CRLM [81]. The latest research showed that the strategy of FOLFOXIRI plus bevacizumab before and after disease progression seemed to be more beneficial to improve the prognosis of patients with metastatic CRC than sequential administration of chemotherapy doublets, in combination with bevacizumab [97]. However, for patients with KRAS WT, there was still controversy when choosing EGFR inhibitor or anti-VEGF combined with chemotherapy [98–101]. In addition, studies showed that the mutational status of patients with metastatic CRC was different in different primary tumor sites [102–104], which could directly affect the response of patients to perioperative chemotherapy and targeted drugs [105, 106]. However, most previous studies focused on the mutational status from the perspective of left and right CRC [107–109]. Recent studies have shown that the mutational status of CRC such as TP53, KRAS, BRAF^V600^, and PIK3CA varied with different primary tumor sites [29]. Based on the importance of mutational status in the treatment for metastatic CRC and the lack of research on different colorectal subsites, it was necessary to further carry out the study of mutational status for different colorectal subsites such as transverse colon including flexures cancer based on previous studies to guide the choice of perioperative chemotherapy for patients with unresectable liver metastasis from transverse colon including flexures cancer.

In this study, the SEER database was used to analyze the effect of PTR on the survival of UTCLM, UHFLM, and USFLM patients, because the SEER database could access more patients than a single institution. However, this study had some limitations: first, although the SEER database provided the scope of colectomy, it did not provide further surgical information, such as laparoscopy or laparotomy, operation time, lymph nodes yields, blood loss, postoperative complications, and so on. These factors may also affect the survival outcomes; second, chemotherapy is one of the important treatment methods for patients with unresectable CRLM. The SEER database did not provide specific chemotherapy regimens, curative time and effect, which may affect the judgment of surgical efficacy to a certain extent; third, since the SEER database did not provide details of the number and size of metastases of individual organ and more details of metastatic organs (such as peritoneum), we were unable to further analyze the effects of different tumor burden on the choice of surgical strategy for patients with UTCLM, UHFLM and USFLM; fourth, although gene mutation status played an important role in guiding surgical strategy, and perioperative chemotherapy and targeted therapy, due to the lack of more detailed information on gene mutation status in the SEER database, we did not further analyze the effects of different gene mutation states on PTR and perioperative chemotherapy for patients with UTCLM, UHFLM and USFLM; finally, there was a selective bias in the retrospective study, for example, the choice of the patients undergoing PTR may be affected by the patient’s functional status, clinical symptoms and signs, degree of metastasis, related complications and so on.

## Conclusion

In summary, we confirmed the different survival of UTCLM, UHFLM, and USFLM patients, and for the first time, we proved that PTR could provide survival benefits for patients with unresectable CRLM from the perspective of colonic subsites of transverse colon, hepatic flexure, and splenic flexure. Besides, PTR may not improve the prognosis of USFLM patients with CEA-negative or tumor size≤5 cm. Our results suggested that surgical procedures such as LCR have no statistically significant prognostic benefits over less aggressive approaches such as SCR for UTCLM, UHFLM, and USFLM patients. For oncologic outcomes, we concluded that SCR seemed an effective surgical procedure for UTCLM, UHFLM, and USFLM. In addition, gene mutation analysis of different colon subsites such as transverse colon including flexures should be considered to guide PTR and perioperative chemotherapy for unresectable CRLM.

## Data Availability

The data supporting the results of this study are available in the SEER 18 regions database [Incidence-SEER 18 Regs Research Data (with additional treatment fields), Nov 2018 Sub (1975–2016 varying)] https://seer.cancer.gov/data/, and can be obtained from the corresponding authors on reasonable request. We firstly logged in to the SEER^*^Stat software with a username of 13521-Nov2019, submitted a data retrieval request, and then we extracted the eligible data after the authorization of the SEER database.

## Abbreviations

SEER: Surveillance, Epidemiology, and End Results
CRC: Colorectal cancer
CRLM: colorectal cancer liver metastasis
OS: Overall survival
CSS: Cause-specific survival
PTR: Primary tumor resection
SCR: Segmental colon resection
LCR: Larger colon resection
UHFLM: Unresectable hepatic flexure cancer liver metastasis
UTCLM: Unresectable transverse colon cancer liver metastasis
USFLM: Unresectable splenic flexure cancer liver metastasis
